# Systematic screening of glycosylation- and trafficking-associated gene knockouts in *Saccharomyces cerevisiae* identifies mutants with improved heterologous exocellulase activity and host secretion

**DOI:** 10.1186/1472-6750-13-71

**Published:** 2013-09-03

**Authors:** Tzi-Yuan Wang, Chih-Jen Huang, Hsin-Liang Chen, Po-Chun Ho, Huei-Mien Ke, Hsing-Yi Cho, Sz-Kai Ruan, Kuo-Yen Hung, I-Li Wang, Ya-Wun Cai, Huang-Mo Sung, Wen-Hsiung Li, Ming-Che Shih

**Affiliations:** 1 Biodiversity Research Center, Academia Sinica, Taipei 115, Taiwan; 2 Molecular and Biological Agricultural Sciences Program, Taiwan International Graduate Program, National Chung-Hsing University – Academia Sinica, Taipei 115, Taiwan; 3 Graduate Institute of Biotechnology, National Chung-Hsing University, Taichung 402, Taiwan; 4 Program in Microbial Genomics, National Chung Hsing University – Academia Sinica, Taipei 115, Taiwan; 5 Agricultural Biotechnology Research Center, Academia Sinica, Taipei 115, Taiwan; 6 Department of Life Sciences, National Cheng Kung University, Tainan 701, Taiwan; 7 Department of Ecology and Evolution, University of Chicago, Chicago IL 60637, USA

**Keywords:** Cellulase production, Glycosylation, Protein secretion

## Abstract

**Background:**

As a strong fermentator, *Saccharomyces cerevisiae* has the potential to be an excellent host for ethanol production by consolidated bioprocessing. For this purpose, it is necessary to transform cellulose genes into the yeast genome because it contains no cellulose genes. However, heterologous protein expression in *S. cerevisiae* often suffers from hyper-glycosylation and/or poor secretion. Thus, there is a need to genetically engineer the yeast to reduce its glycosylation strength and to increase its secretion ability.

**Results:**

*Saccharomyces cerevisiae* gene-knockout strains were screened for improved extracellular activity of a recombinant exocellulase (PCX) from the cellulose digesting fungus *Phanerochaete chrysosporium*. Knockout mutants of 47 glycosylation-related genes and 10 protein-trafficking-related genes were transformed with a PCX expression construct and screened for extracellular cellulase activity. Twelve of the screened mutants were found to have a more than 2-fold increase in extracellular PCX activity in comparison with the wild type. The extracellular PCX activities in the glycosylation-related *mnn10* and *pmt5* null mutants were, respectively, 6 and 4 times higher than that of the wild type; and the extracellular PCX activities in 9 protein-trafficking-related mutants, especially in the *chc1*, *clc1* and *vps21* null mutants, were at least 1.5 times higher than the parental strains. Site-directed mutagenesis studies further revealed that the degree of *N*-glycosylation also plays an important role in heterologous cellulase activity in *S. cerevisiae*.

**Conclusions:**

Systematic screening of knockout mutants of glycosylation- and protein trafficking-associated genes in *S. cerevisiae* revealed that: (1) blocking Golgi-to-endosome transport may force *S. cerevisiae* to export cellulases; and (2) both over- and under-glycosylation may alter the enzyme activity of cellulases. This systematic gene-knockout screening approach may serve as a convenient means for increasing the extracellular activities of recombinant proteins expressed in *S. cerevisiae*.

## Background

Bioethanol derived from cellulosic biomass may constitute an environmentally-friendly replacement for fossil fuel in the future [1-3]. Several kinds of bioprocesses have been proposed for the conversion of biomass into cellulosic ethanol such as separate hydrolysis and fermentation (SHF), simultaneous saccharification and fermentation (SSF), simultaneous saccharification and co-fermentation (SSCF), and consolidated bioprocessing (CBP). Among these, CBP seems to be the most promising because of its potential high efficiency and low cost [4]. To achieve CBP, a microbe that is capable of producing secretory cellulases is needed to digest cellulose into a suitable carbon source to produce ethanol by fermentation.

*Saccharomyces cerevisiae* is the most widely-used microorganism for fermentation because of its high ethanol conversion rate. Over the past decade, a number of cellulases have been discovered from animal guts, forest fungi and plants, some of which were successfully expressed in *Pichia pastoris*[5-22]. Several patented or commercial extracellular cellulases were derived from *Trichoderma* species [23-26]. However, to establish a CBP platform using *S. cerevisiae*, the host needs to be modified to express and secrete recombinant cellulases more efficiently [1,4,27,28].

Post-translational modifications in *S. cerevisiae* affect the functions of recombinant proteins. For example, *N*-glycosylation in yeast is of the high-mannose type [29-31] and such *N*-glycosylation of a recombinant cellulase was found to reduce its activity and enhance its non-productive binding on cellulose [32]. On the other hand, *Trichoderma reesei* benefits from overexpression of a *S. cerevisiae* glycosylation gene (*DPM1*) to increase its own cellulase activity [33]. Therefore, understanding how glycosylation affects the properties of foreign proteins in yeast cells is helpful for the development of *S. cerevisiae* as a production host for heterologous proteins.

One way to achieve CBP is to express and secrete all of the three essential cellulases, namely endocellulase, exocellulase and beta-glucosidase, by *S. cerevisiae* for direct digestion of cellulosic materials [19,34,35]. Exocellulase is often considered the rate-limiting enzyme during cellulosic degradation. The importance of the exocellulase activity has been demonstrated in several studies [24,26,36]. It has been reported that a white-rot fungus *Phanerochaete chrysosporium* showed more cellulase production and higher activities than three *Trichoderma* spp. in agricultural waste [37]. Unlike *T. reesei*, *P. chrysosporium* produces more different types of CBHI-like cellobiohydrolases [38], and secretes many exocellulases, especially the glycoside hydrolase family 7 (GH7) exocellulases during cellulosic degradation. Omics approaches [39-41] and functional studies [42-46] have shown that many potential cellulases exist in this white-rot fungus. From the genome sequence of *P. chrysosporium*, we selected several putative GH7 genes [47] for expression in *S. cerevisiae*. We found that instead of secreting the recombinant cellulase into the extracellular space, most of the transformants accumulate the heterologous cellulase inside the cells. After activity screening, we chose the GH7 gene with the highest exocellulase activity, designated as PCX, for further investigation.

Several studies have shown that it is possible to improve the secretion efficiency of recombinant proteins expressed in yeast by manipulating the cellular protein trafficking and glycosylation pathways (for reviews, see [48-52]). To examine the effects of changes in glycosylation- and protein trafficking-associated genes on extracellular PCX activity, we transformed the PCX cDNA into yeast knockout (KO) strains of 47 glycosylation-related and 10 protein trafficking-associated genes to screen for those with increased cellulase activity. Our results showed that knockouts of two glycosylation-related genes (*MNN10* and *PMT5*) and one Golgi-to-endosome transport gene (*VPS21*) increased extracellular exocellulase activity up to 4 to 6 fold. For comparison, we also expressed four cellulase genes from three other fungi, an endocellulase (EgIII) and an exocellulase (CBHI) from *T. ressei*, an endocellulase (Cen1) from *Irpex lacteus*, and a beta-glucosidase (BglI) from *Aspergillus niger* in *S. cerevisiae*.

## Results and discussion

### Low level of secretion of recombinant cellulases in *S. cerevisiae*

**Figure 1:**
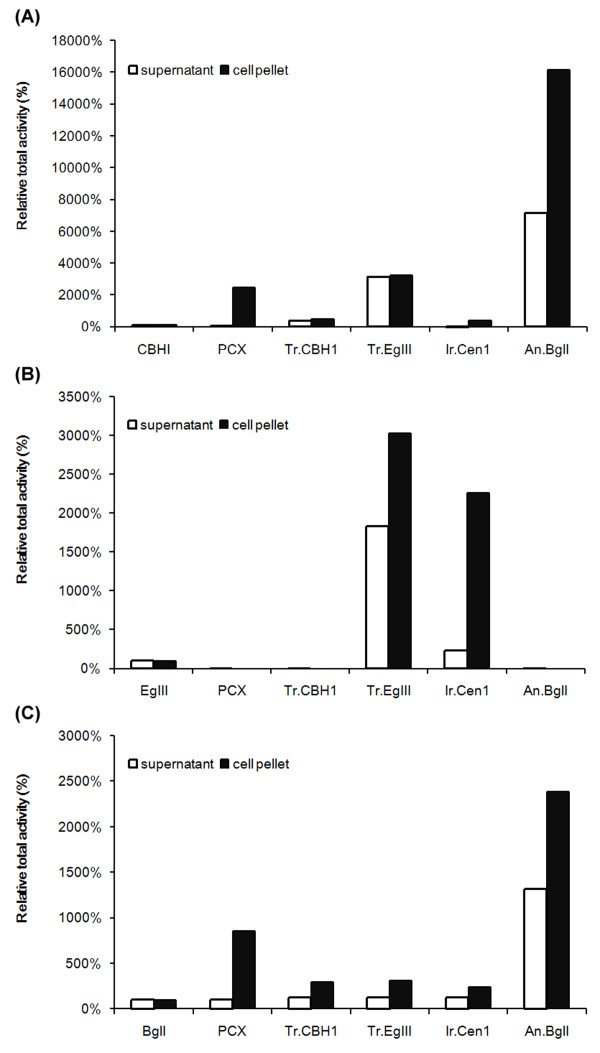
**Relative total activities of recombinant cellulases expressed in *S. cerevisiae*. (A)** 4-methylumbelliferyl-β-D-cellobiose (4-MUC assay) for exocellulase activity with CBH I as a relative specific activity marker (100%). **(B)** Dye-carboxylmethy cellulose (dye-CMC) assay for endoglucanase activity with EgIII as the relative specific activity marker (100%). **(C)***p*-nitrophenyl-b-D-glucopyranoside (*p*NPG) assay for beta-glucosidase activity with BglI as the relative specific activity marker (100%). The expression plasmid and related information are described in the Methods section. The 4-MUC, Dye-CMC and *p*NPG assay methods were as described in [21].

Exocellulase PCX from *P. chrysosporium*, endocellulase EgIII and exocellulase CBHI from *T. ressei*, and endocellulase Cen1 from *I. lacteus* were all successfully expressed in *S. cerevisiae* BY4741 strain. However, when the alpha factor signal peptide was used as the secretion signal, none of these heterologously expressed cellulases could be secreted from yeast cells. Lower cellulase activity was observed in protein extracted from the supernatant of the cell culture than in protein extracted from cell pellets (Figure 1). It has been suggested that over-glycosylation reduces the activity of recombinant cellulases and may also reduce their secretion ability in yeast [53]. Moreover, the Golgi-to-endosome transportation pathway may also interfere in protein exocytosis because the Golgi-endoplasmic reticulum system is responsible for the degradation and detoxification of heterologous proteins [54]. Therefore, we investigated whether mutations in the glycosylation pathway or in the Golgi-to-endosome trafficking pathway affected the secretion of heterologous proteins or their cellulase activity.

### Deglycosylation increases extracellular PCX cellulase activity

**Figure 2:**
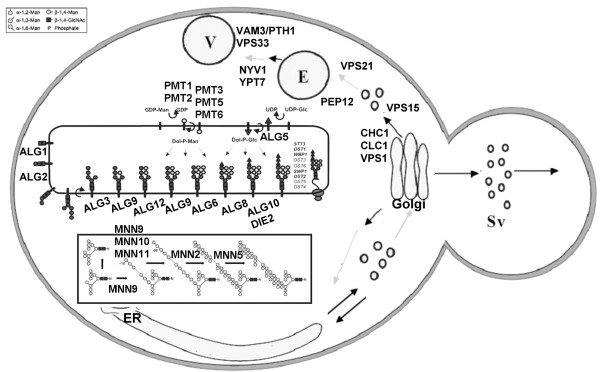
Major gene families involved in the glycosylation and protein transport pathways in yeast.

A survey of *S. cerevisiae* BY4741 gene knockout collections (Open Biosystems) identified more than 70 viable strains with knockout mutations in genes related to glycosylation and protein trafficking. We successfully transformed the PCX coding sequence into 57 single-gene-knockout strains; the 57 genes included 47 glycosylation-related genes, including those from the major *ALG*, *OST*, *MNN*, *PMT* gene families, and 10 trafficking-associated genes involved in Golgi-to-endosome-vacuole transport (Table 1 & Figure 2). We then used the 96-well plate screening method to test candidate transformants for increased total extracellular cellulase activities using the 4-methylumbellifery-β-D-cellobioside (4-MUC) assay (see Table 1, 96-well screening 4-MUC assay). The extracellular PCX activities of 22 of the 47 glycosylation-related gene knockout strains and all except one of the Golgi-to-endosome transport pathway mutants increased at least 1.3-fold relative to expression in the wild-type strain. To ensure that the screening results were reliable, we further condensed the supernatants from 50-ml cell culture and reanalyzed their activities. Interestingly, the extracellular PCX cellulase expressed in the glycosylation-related *mnn10* and *pmt5* null mutants showed 6.0- and 4.3-fold increases in cellulase activity, respectively, compared to expression in the wild-type strain (see Table 1, condensed sample 4-MUC assay). We also tested the NpaBGS beta-glucosidase isolated from the *Neocallimastix patriciarum* W5 strain [55] with 40 of the 57 single-gene-knockout BY4741 strains and obtained similar results (Table 1).

**Table 1:** **Screening of extracellular PCX cellulase activity of knockout and original *S. cerevisiae *BY4741 strains by 4-MUC assay (recombinant/original ratio)**

***Gene null mutant***	***ORF name***^**a**^	***96-well screening 4-MUC assay***	***Condensed sample 4-MUC assay (o/n OD***_***600***_***)***
Protein-trafficking genes:			
*chc1∆::KanMX*	YGL206C	2.5	2.4 (2.8)
*clc1∆::KanMX*	YGR167W	3.8	2.6 (3.1)
*nyv1∆::KanMX*	YLR093C^f^	1.5	1.7 (5.7)
*pep12∆::KanMX*	YOR036W	1.9	2.6 (2.9)
*vam3∆::KanMX*	YOR106W	1.6	NA
*vps1∆::KanMX*	YKR001C	1.2	NA
*vps15∆::KanMX*	YBR097W	1.9	1.3 (4.2)
*vps21∆::KanMX*	YOR089C^f^	3.3	6.5 (6.1)
*vps33∆::KanMX*	YLR396C	1.5	0.9 (5.5)
*ypt7∆::KanMX*	YML001W^b^	1.7	1.0 (5.1)
Glycosylation-related genes:			
*aga2∆::KanMX*	YGL032C	0.9	NA
*alg3∆::KanMX*	YBL082C^c^	0.5	NA
*alg5∆::KanMX*	YPL227C^c^	0.7	NA
*alg6∆::KanMX*	YOR002W^c^	0.8	NA
*alg8∆::KanMX*	YOR067C^c^	1.5	NA
*alg9∆::KanMX*	YNL219C^c^	1.5	NA
*alg12∆::KanMX*	YNR030W^c^	1.7	NA
*anp1∆::KanMX*	YEL036C^c^	1.8	NA
*csg2∆::KanMX*	YBR036C	1.0	NA
*cwh41∆::KanMX*	YGL027C^c^	0.1	NA
*cwh43∆::KanMX*	YCR017C	1.3	NA
*die2∆::KanMX*	YGR227W^c^	1.5	NA
*gda1∆::KanMX*	YEL042W	1.6	NA
*gnt1∆::KanMX*	YOR320C	1.2	NA
*gon7∆::KanMX*	YJL184W	0.1	NA
*gtb1∆::KanMX*	YDR221W^d,e^	2.7	0.0 (12.6)
*hoc1∆::KanMX*	YJR075W	0.9	NA
*hor7∆::KanMX*	YMR251W-A	1.4	NA
*ktr1∆::KanMX*	YOR099W^d^	0.8	NA
*ktr2∆::KanMX*	YKR061W^d,f^	0.9	NA
*ktr3∆::KanMX*	YBR205W^d^	1.0	NA
*ktr4∆::KanMX*	YBR199W^d^	1.0	NA
*ktr5∆::KanMX*	YNL029C^c^	1.0	NA
*ktr6∆::KanMX*	YPL053C^d^	1.3	NA
*ktr7∆::KanMX*	YIL085C^d^	1.0	NA
*mnl1∆::KanMX*	YHR204W^d^	1.2	NA
*mnn2∆::KanMX*	YBR015C^c^	1.3	0.8 (3.8)
*mnn5∆::KanMX*	YJL186W^f^	1.9	3.0 (5.6)
*mnn9∆::KanMX*	YPL050C^c,f^	2.2	3.8 (5.5)
*mnn10∆::KanMX*	YDR245W	2.5	6.0 (3.8)
*mnn11∆::KanMX*	YJL183W^c^	2.0	4.5 (3.7)
*mns1∆::KanMX*	YJR131W^d^	0.5	NA
*mnt2∆::KanMX*	YGL257C^d^	1.2	NA
*mnt3∆::KanMX*	YIL014W^d,e^	2.5	1.7 (5.2)
*mrl1∆::KanMX*	YPR079W^f^	1.6	NA
*ost3∆::KanMX*	YOR085W^c^	1.1	NA
*ost4∆::KanMX*	YDL232W^c,f^	1.6	NA
*ost5∆::KanMX*	YGL226C-A^c^	0.9	NA
*ost6∆::KanMX*	YML019W^c,f^	1.8	NA
*pmt1∆::KanMX*	YDL095W^c^	3.1	3.2 (4.7)
*pmt2∆::KanMX*	YAL023C^c^	5.3	1.8 (4.7)
*pmt3∆::KanMX*	YOR321W^c^	1.1	1.0 (4.4)
*pmt5∆::KanMX*	YDL093W^c^	2.1	4.3 (6.3)
*pmt6∆::KanMX*	YGR199W^c^	0.8	NA
*ynd1∆::KanMX*	YER005W^f^	1.1	NA
*yur1∆::KanMX*	YJL139C^f^	2.0	2.8(4.5)
*yjr061w∆::KanMX*	YJR061W	1.0	NA

^a^YGL038C, YLR240W, YGL095C knockout strains failed to be transformed with PCX.

^b^Enhancement of Ctcel8A endocellulase activity [52].

Enhancement^c^ and decrease^d^ of Cel8Aenz endocellulase activity [48].

^e^Detectable NpaBGS beta-glucosidase activity as screened by *p*NPG assay.

^f^The null mutant could grow in SC-URA medium with 1% cellobiose when the NpaBGS beta-glucosidase gene is functional inside.

The 4-MUC activity ratio is the average PCX activity of the three highest expressing recombinants (single-gene-KO strain) to the average original PCX activity (BY4741 strain).

Our 4-MUC analyses also showed an increase in extracellular PCX activity in the *ALG* and *OST* null mutants (Table 1). It is known that the *ALG* and *OST* family genes play roles in the initiation of the addition of *N*-glycans to proteins and the elongation of mannose in the endoplasmic reticulum [56,57]. *ALG8*, *ALG9*, *ALG12*, *DIE2*, *OST4 and OST6* are downstream glycosylation pathway genes responsible for *N*-mannose and *N*-glucose elongation. Extracellular PCX activity was also increased in *MNN* and *PMT* mutants. In the Golgi, *MNN* family proteins add *N*-mannose to complete *N*-linked glycosylation, while the *PMT* family genes encode enzymes elongating oligomannoses for assembling *O*-glycosylation [31]. A previous study indicated that the disruption of the *MNN10* gene enhanced protein secretion in yeast [53]. Our 4-MUC analysis also showed increases in extracellular PCX activity in *mnn2*, *mnn5*, *mnn9*, *mnn10* and *mnn11* null mutants (Table 1). These genes are also downstream glycosylation pathway genes responsible for high *N*-mannose glycosylation (adding more than 10 mannose polymers). Extracellular PCX activity was also increased in the *pmt1*, *pmt2*, *pmt3* and *pmt5* null mutants. Previous studies have also suggested that mutations in *MNN* and *PMT* can increase endocellulase production in *S. cerevisiae*[48]. In summary, these results suggest that knocking out *O*- and *N*-glycosylation-related genes can indeed increase the total cellulase activity of recombinant extracellular cellulase PCX.

### Blocking Golgi-to-endosome transport increases extracellular PCX cellulase activity

**Figure 3:**
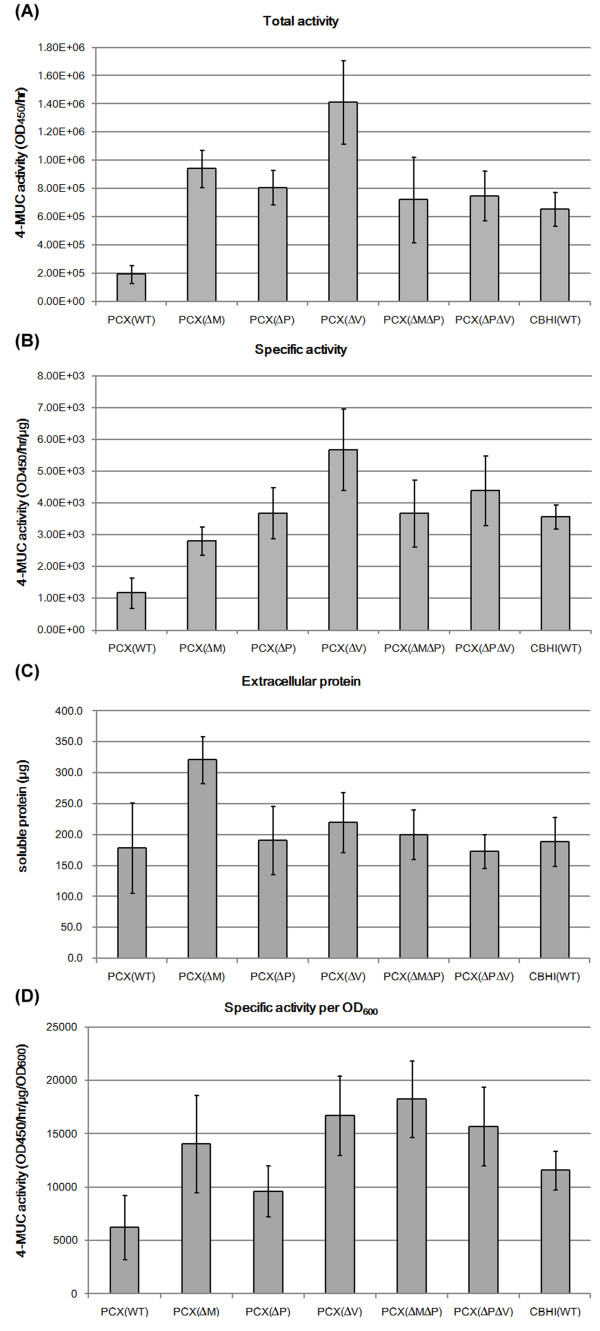
**Extracellular PCX activity in 50 ml supernatants of different hosts as measured by 4-MUC assay. (A)** Total extracellular activity; **(B)** specific extracellular activity; and **(C)** total extracellular proteins. WT: BY4741; *∆*M: *mnn10* null mutant*;∆*V: *vps21* null mutant; *∆*P: *pmt5* null mutant; *∆*M*∆*P: *mnn10∆pmt5∆* double knockout strain; *∆*P*∆*V: *pmt5∆vps21∆* double knockout strain; CBHI: exocellulase of *Trichoderma ressei*.

In yeast cells, heterologously expressed proteins are often transported to endosomes for degradation via *VPS* family proteins and others [58]. Our results showed that extracellular PCX activity was increased (more than 1.5-fold in 96-well screening) in 9 of the 10 Golgi-to-endosome-related gene knockout mutants, especially in the *chc1*, *clc1* and *vps21* null mutants (Table 1). The supernatants from 50-ml cell culture were condensed and re-analyzed for their activities. The *vps21* null mutant had the highest extracellular PCX cellulase activity, 6.5 times higher than that of the parental strain. Chc1p and Clc1p are subunits of the clathrin protein and can form clathrin-coated vesicles for intracellular protein transport, while Vps21p is a part of the CORVET complex required for vesicle transport between the vacuole and endosome (Figure 2). A recent study also found that inhibition of vesicle sorting to vacuoles could increase endocellulase production in *S. cerevisiae*[52]. These results imply that blocking the endocytosis pathway can indirectly influence *S. cerevisiae* to export PCX through the vesicle exocytosis pathway. However, total extracellular proteins only increased slightly in the *vps21* null mutant (Figure 3). Therefore, other unknown factors may influence the extracellular PCX activity.

### Multiple-gene-knockout strains do not show further improvement in PCX activity and secretion

Next, we constructed double gene knockout strains of *MNN10*, *PMT5* and *VPS21* to examine whether a combination of mutations would further increase the cellulase activity and secretion of the PCX enzyme. The generation times of the single- and double-gene knockout strains were shorter than that of the parental BY4741 strain (Additional file 1). Among these strains, the highest extracellular PCX activity was recorded after incubation for 24 hours (Additional file 2). In addition, the growth rate of each mutant might be less important for extracellular PCX activity. For example, the *vps21* null mutant had higher enzyme activity than the *pmt5* null mutant even though the *pmt5* null mutant had a shorter doubling time and higher saturation OD_600_ (Additional file 1). Thus, in further analyses we compared the secreted PCX activity in single- and double-gene-knockout strains in 24-hour cultures (Figure 3).

The data showed that the *mnn10* and *vps21* null mutants had a higher extracellular PCX activity than those of the double-gene knockout mutants. A previous study [32] reported that the Mnn10 protein elongated *N*-glycan by adding mannose to the recombinant proteins and could reduce the heterologous protein activity in yeast. In contrast, Figure 3 shows that lack of the *MNN10* gene (i.e., deglycosylation) could increase the total activity of extracellular PCX and the secretion of extracellular proteins. We only found significantly more total extracellular protein abundance in the *mnn*10 null mutant but not in the other mutants examined, so we suggest that yeast lacking the *MNN10* gene is suitable to raise the yield of a heterologous protein expressed in a *S. cerevisiae* host system (Figure 3). Second, PMT5p functions in the addition of *O*-linked glycans to amino acids. Our results consistently showed that the *PMT5* mutation, which might result in less *O*-glycosylation, could also increase the total activity of extracellular PCX. Extracellular protein secretion did not increase significantly in the *pmt*5 null mutant, but the total cellulase activity still increased, suggesting that a reduction in *O*-glycosylation increases cellulase activity but may not affect protein secretion. Third, Vps21 is a key player in Golgi-to-vacuole transport. Our results showed that the total extracellular PCX activity was also increased in yeast that lacks Vps21 protein. Among the double gene knockout strains, the *mnn10*∆*vps21∆* knockout strain had very slow growth to produce PCX protein. This double-gene knockout mutant might down-regulate protein secretion, extracellular PCX activity, or cell growth. Therefore, such mutants are probably not suitable for application in cellulosic ethanol production. Furthermore, the *pmt5*∆*vps21*∆ or *mnn10*∆*pmt5*∆ knockout strains increased the total extracellular cellulase activity but also showed slower growth rates than single-gene knockout strains. These results suggest that the increase in extracellular cellulase activity in the *mnn10* null mutant is mainly due to an increase in cellulase protein secretion, not due to protein activity itself, whereas the increase in *pmt*5 and *vps21* null mutants may be mainly due to protein post-translational modifications and/or disordered intracellular trafficking. Together, these results suggest that single-gene knockout strains may be more suitable for bioethanol applications.

### Protein modification differences among recombinant cellulases in null mutants

**Figure 4:**
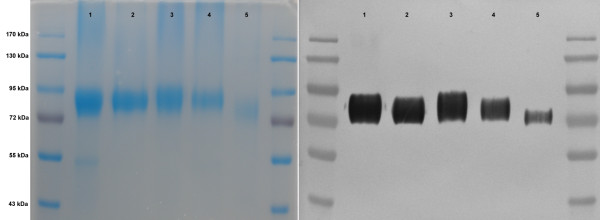
**Extracellular His-tagged PCX from different hosts.** SDS-PAGE, left, 10 μg per lane, and western blot, right, 1 μg per lane of extracellular his-tagged PCX from different hosts. From left to right: lane 1, PCX from the BY4741 strain; lane 2, PCX from the *mnn10* null mutant; lane 3, PCX from the *vps21* null mutant; lane 4, PCX from the *pmt5* null mutant; and lane 5, PCX from the *alg1-1* mutant. The protein marker is the Fermentas PageRuler Pre-stained Protein Ladder.

An increase in extracellular cellulase activity results from changes in glycosylation and/or extracellular secretion. PCX has three potential *N*-glycosylation sites in the catalytic domain and five putative *O*-glycosylation sites in the cellulose-binding domain. To assess the effect of glycosylation on extracellular PCX activity, we compared the effects of abolishing gene function at earlier and later steps in the glycosylation pathway. We introduced the PCX gene into the *alg1-1* mutant (*ALG1)* which contains defects in asparagine-linked glycosylation [59]. The *ALG1* gene functions in elongating *N*-glycan after the formation of the first three glycans (MansGlcNac2) at the *N*-glycosylation site of a protein (Figure 2; [60]). The purified PCX in the *alg1-1* mutant showed a significant shift in molecular weight, while the remaining null mutants (e.g., *mnn10∆::KanMX*) had slightly different SDS-PAGE mobility patterns, except *vps21∆::KanMX* (Figure 4). However, the PCX activity in the *alg1-1* mutant was even lower than that in the knockout strains implying that the modified glycans still played an important role in PCX activity (Additional file 3). Furthermore, the *MNN10* mutant had the highest level of extracellular protein secretion among the knockout strains. A previous study suggested that disruption of the *MNN10* gene increases protein secretion in yeasts due to the change in the structure of glycoproteins in the yeast cell wall which affects the release of secretory proteins into the media at the last stage of protein secretion [53]. However, the *MNN10* gene may be essential to increase yeast tolerance of ethanol-induced stress [61].

We also found that the *pmt5* null mutant, which encodes an *O*-mannosyltransferase, resulted in an increase in extracellular PCX activity (Table 1). The PMT family proteins manage *O*-glycosylation as a sorting determinant for cell surface delivery [62], and protein *O*-glycosylation is essential for cell wall rigidity and cell integrity as well as protein modification in *S. cerevisiae* and *T. reesei*[63,64]. Herein, our results point to the possibility that the *PMT5* mutant might increase the extracellular PCX activity and therefore might also play a role in the protein secretory process.

### *N*-glycosyation site-mutagenesis influences the activity of a recombinant cellulase

**Figure 5:**
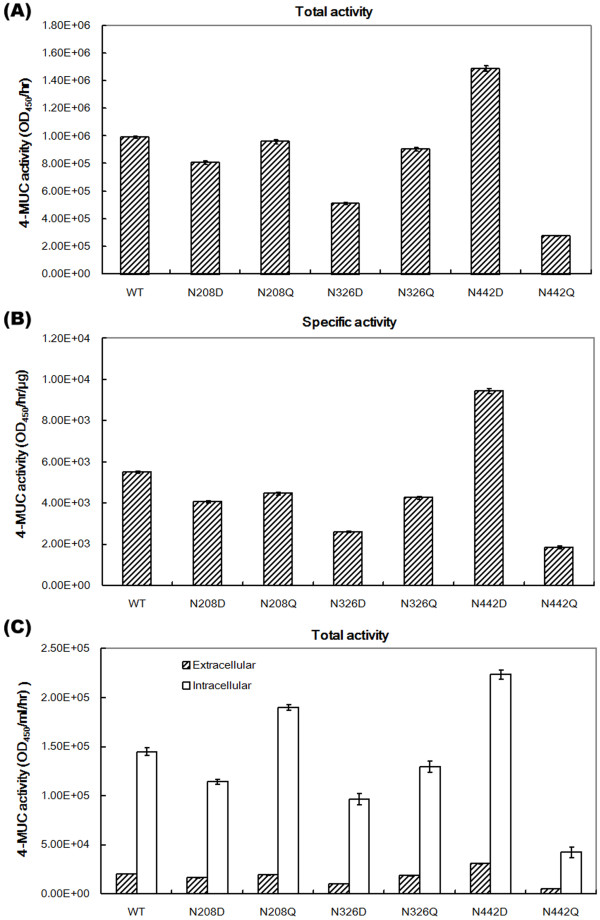
**Extracellular PCX activity is improved by site-mutagenesis of putative *N*-glycosylation amino acids. (A)** Total extracellular activity; **(B)** specific extracellular activity; and **(C)** total activity in extracellular and intracellular soluble proteins.

We next conducted site-directed mutagenesis of putative *N*-glycosylation sites of PCX to examine their possible effects on the extracellular PCX activity (Figure 5A,B). Peptide-N-Glycosidase F (PNGaseF) can cleave high mannose and complex oligosaccharides from N-linked glycoproteins. The deglycosylated PCX proteins treated with PNGaseF (Additional file 4) only showed a statistically significant difference in 4-MUC activity between the *vps*21 null mutant and the wild-type BY4741; the previous two strains added more *N*-glycans to the expressed PCX than the other two null mutants (*pmt5∆* and *mnn10∆*) tested. These results suggest that mutations of the *O*- or *N*-glycosylation sites may influence the extracellular PCX activity.

Protein modeling of cellobiohydrolase 58 based on X-ray data (PDB code 1Z3V) revealed that the N208 site is located near the cellulose-binding tunnel and the other two sites (N326 and N442) are located on the PCX protein surface (Additional file 5). *N*-glycosylation may thus interfere with substrate binding to PCX or conformation structure and influence PCX activity. Therefore, we replaced the three potential *N*-glycosylation amino acid sites (positions N208, N326, and N442) of PCX with aspartate (D) or glutamine (Q). These mutated PCX genes were transformed into yeast to determine their effects on extracellular PCX cellulases. The extracellular activities of all the mutated PCX cellulases, except the N442D mutant, were lower than those of the wild-type PCX cellulase (Figure 5). The N442Q mutant showed significantly lower extracellular PCX activity. The total and specific extracellular activities in the N442D mutant, however, were higher than those in wild-type PCX. These results suggest that the individual mutations on the PCX gene also affect PCX activity.

We also extracted the intracellular soluble proteins from cell pellets to evaluate the secretary efficiencies of the six mutants - N208D, N208Q, N326D, N326Q, N442D, N442Q (Figure 5C). The PCX extracellular and intracellular soluble protein activities of the N208D, N326D, and N442Q mutants were much lower than the other mutants. The N442Q mutant showed the most significant reduction in extracellular and intracellular activities. In contrast, the N208Q and N442D mutants showed higher intracellular and higher extracellular activity than wild-type PCX (Figure 5). Furthermore, when the mutated PCX gene was manipulated into the *mnn10*, *pmt5*, and *vps21* null mutants, the extracellular activities in the three mutants were still higher than the wide-type strain as in Figure 3 (data not shown). Thus, the three mutants are indeed suitable hosts for extracellular PCX protein production. In short, the combination of N442D site-mutated PCX protein in *vps21* null mutants may exhibit the highest extracellular PCX activity.

### Comparison of null mutants that enhance different cellulase protein production in *S. cerevisiae*

Our study complements two recent studies [48,52]. Kitagawa *et al.* (2011) reported 55 *S. cerevisiae* knockout strains that exhibited enhanced Ctcel8A endocellulase activity. The *vps3∆* and *vps16∆* strains, which have deletions in genes encoding components of the class C core vacuole/endosome tethering (CORVET) complex, also exhibited enhanced beta-glucosidase activity of Ctcel8A proteins. Both Vps3p and Vps21p are major components of the same CORVET complex. Thus, inhibition of the endocytosis pathway indeed could improve heterologous cellulase production. In a second study, Suzuki *et al.* (2012) screened for enhancement of the endocellulase Cel8Aenz using 44 protein glycosylation mutants. The mutations in *ALG*, *PMT* and *MNN* family genes also improved the Cel8Aenz production in *S. cerevisiae*. These results and our results shown in Table 1 indicate that mutations such as *mnn9∆, ost4∆, ost6∆* that cause reduction of *N*-glycosylation abilities of the yeast host can increase the heterologous celluase activities. Furthermore, *O*-glycosylation deficiency, such as seen in the *pmt1∆, pmt2∆, pmt5∆* mutants, could also improve heterologous cellulase production.

Our screening method (adopted from [21]) could quantify and sensitively detect the total activity of exoglucanase easily, while the plate method [52] was not sensitive enough to apply in exoglucanse screening. The systemic analyses using the yeast deletion strain collection allowed us to identify genes that, when mutated, could increase the efficiency of cellulase production.

## Conclusions

Systematic screening and analysis revealed that some *S. cerevisiae* glycosylation and protein-trafficking pathway gene-knockout mutants improve extracellular cellulase activity and undergo functional changes during heterologous protein synthesis in yeast. It is known that *N*-glycosylation in yeast is of the high-mannose type and that increased *N*-glycosylation of recombinant cellulases reduces their activity [29,32-34]. Our screening of gene-knockout mutants of the glycosylation pathway showed that the activity of recombinant PCX cellulase increased significantly in some mannose-glycosylation gene knockout strains such as *mnn9*, *mnn10*, *mnn11* and *mnt3*. Heterologous PCX cellulase activity was affected by not only the high-mannose glycosylation pattern, but also the activity of intracellular trafficking systems of *S. cerevisiae*. Systematic screening of knockouts of intracellular trafficking-associated genes showed that the activity of extracellular PCX cellulase increased significantly when some intracellular trafficking-associated genes such as *CHC1*, *CLC1*, and *VPS21* were inactivated. *O*-glycosylation associated genes, such as *PMT1*, *PMT2* and *PMT5*, which are involved in post-translational modification of the endoplasmic reticulum, also influenced the cell wall rigidity for the secretion of the PCX cellulase protein, potentially posing an obstacle to the efficient use of *S. cerevisiae* as host in a consolidated bioethanol production system. Our systematic gene knockout screening approach has proven to be useful to test the secretion capability and cellulase activity of proteins heterologously expressed in *S. cerevisiae*. In the future, this approach can be further modified as an automatic high-throughput platform to speed up the screening process to find *S. cerevisiae* mutant strains that may be suitable for use in bioprocessing.

## Methods

### White-rot fungial cDNA preparation

Cells of *P. chrysosporium* (ATCC 32629) were grown for 5 days on PDA medium (diced potatoes 200.0 g/l, glucose 20.0 g/l, agar 15.0 g/l) at 25°C [65]. About thirty squares (5 mm × 5 mm) of mycelium mat were inoculated into 100 ml defined medium (PD) with 1% rice straw and grown at 25°C with shaking at 170 rpm for 5–7 days. The filtered cell culture mass was ground in liquid nitrogen, and total RNA was extracted using the hot acid phenol-chloroform method [66] and quantified on a spectrophotometer. The total RNA was converted into cDNA using the SuperScript™ II Reverse Transcriptase Kit (Invitrogen, Carlsbad, CA). The reaction contained total RNA (4 μg), oligo(dT) primer, BD SMART II A oligo (Clontech, Mountain View, CA, USA), 4 μL of 5× RT buffer, 10 mM DTT, 0.5 mM each of deoxyribonucleotide triphosphate and 10 U of Superscript II RNase H Reverse Transcriptase (Invitrogen). The PCX coding sequence was then cloned into pGEM-T easy plasmid (Promega, Madison, WI).

### Plasmid construction and gene cloning

The pRS426-GD plasmid was constructed with GAPDH 5’-promoter (upstream 500 bp) with/without an alpha factor sequence and 3’-terminator region (downstream 300 bp) of the *S. cerevisiae* genomic sequence into the pRS426 plasmid backbone for cellulase gene expression (Additional file 6). The His-tagged cellulase construct was cloned into pGEM-T Easy Vector System (Promega) and confirmed by diagnostic PCR and sequencing. The confirmed cellulase cDNAs were then cloned into the expression vector pRS426-GD via restriction enzyme (EcoRI + SmaI) digestion and denatured-renatured techniques [67].

### Knockout yeast strains

The yeast strain BY4741 (*MATa his3∆1 leu2∆0 met15∆0 ura3∆0*) is a descendant of S288C. The single gene knockout strains (*gene∆::KanMX*) in Table 1 were derived from BY4741 by Open Biosystems Company cat# YSC1021 [68]. All the selected genes are listed in Table 1. The *alg1-1* (*MATa alg1-1 ura3-52 mal gal2*) (ATCC 208304) is an *ALG1* deficient mutant for glycosylation study [59], which is required for adding three glycans (MansGlcNac2) directly onto the protein *N*-glycosylation site (see Figure 2). To construct the double gene-knockout strains, the *MATa*-type *MNN10*, *VPS21* and *PMT5* knockout strains were switched into the *MAT*α-type by introducing the pGAL-HO plasmid [69]. Two knockout strains with *MATa* and *MAT*α, respectively, were mated with each other to create the diploid hybrids, which were then further sporulated into haploid segregants. The segregants for double-gene knockout clones were selected by PCR following the manufacturer’s instructions.

### Yeast transformation

Yeast strains were grown in YPAD medium and harvested at the mid-log phase. Overnight yeast cultures were used to prepare the starting cultures with OD_600_ = 0.1 and competent cells were grown in YPAD media at 30°C with 250 rpm shaking until OD_600_ = 0.8-1.0, and harvested. The plasmid transformation was performed by the LiAc/SS Carrier DNA/PEG method [70] and successful transformants were selected on a SC-URA plate.

### Detection of PCX cellulase activity by 4-MUC assay

Ten colonies of PCX transformants from each strain were randomly selected and cultured in 96 deep-well plates with 500 μl SC-URA medium at 30°C for 1–3 days. For cellulase activity screening, 50 μl supernatants were transferred into 96-well plates, mixed with 1 mg/ml 4-MUC substrates (Sigma-Aldrich, St Louis, MO), and incubated for 24 hours at 30°C at suitable pH (Additional file 7). The OD_365_/OD_450_ (excitation/emission) reads were measured by SpectraMax M2 Microplate Readers to indicate higher exocellulase activity transformants. In addition, to confirm the 4-MUC screening results, at least three colonies with a higher 4-MUC activity of each gene were diluted into OD_600_ = 0.1 and grown in 50 ml SC-URA medium at 30°C for 24 hours. After 24 hours in the stationary phase, most of the cell density is saturated except the strains with slow growth rates. The total PCX enzyme activities are more stable in the stationary phase (Additional file 2). The condensed supernatants were assayed again.

We then selected the colonies with the highest expression to evaluate the total extracellular cellulase activity in the condensed supernatants by 4-MUC assay. In *S. cerevisiae*, the copy number of the pRS426 plasmid is about 20 per haploid cell. The copy number variations did not always correlate to the total 4-MUC activity among different hosts. For example, the *vps21* null mutant with similar relative copy numbers showed different 4-MUC activity (Additional file 8). To avoid activity variation due to different cellulase gene expression levels or loss of plasmid copies, we screened and selected the three colonies with the highest activity within each experimental culture for further analysis and performed at least three biological replicates for each experiment. The overnight cultures of these candidates were diluted into OD_600_ = 0.1 and grown in 50 ml SC-URA medium at 30°C for 24 hours. The 50 ml supernatants were then dialyzed and condensed 100-fold by a Vivaspin 20 10 K NMWL (MWCO) concentrator (Sartorius, Goettingen, Germany). Enzyme activities were measured by the 4-MUC assay [21]. The soluble protein concentration was measured with the Protein Assay reagent (500–0006; Bio-Rad Laboratories, Hercules, CA, USA). The reaction time of the 4-MUC assay was 24 hours for condensed supernatant and one hour for cell pellet-extract at 30°C. In addition, we chose the total cellulase activity to evaluate suitable strains for CBP application because the strains with higher cell density usually have better total cellulase activity.

### Protein purification

Cell lysates of PCX transformants of the wild-type and knockout strains were harvested by centrifugation and the supernatants were dialyzed with a binding buffer [8 M urea, 0.1 M NaH_2_PO_4_, 0.01 M Tris-Cl, pH 8.0]. Purification was achieved by a Ni Sepharose 6 Fast Flow column (GE Healthcare, Taipei, Taiwan). The column was washed with wash buffer [8 M urea, 0.1 M NaH_2_PO_4_, 0.01 M Tris-Cl, pH 6.3] and the recombinant PCXs were then eluted with an elution buffer [8 M urea, 0.1 M NaH_2_PO_4_, 0.01 M Tris-Cl, pH 4.5].

### Western blot

The purified recombinant PCX from wild-type and knockout strains were run on an 8% SDS–polyacrylamide gel and the proteins were then transferred onto a PVDF (GE Healthcare) in an electroblotting buffer (25 mM Tris–HCl, 192 mM glycine, 20% methanol) at 400 mA for 1 h at 4°C. The membrane was blocked with a non-fat milk buffer [5% blocking reagent in TBS buffer (125 mM NaCl, 25 mM Tris, pH 8.0)] at 4°C overnight. A His-tag monoclonal antibody (1:3000) was used as the primary antibody, and the AP-α-mouse (goat) mouse antibody (1:2000) was used as the secondary antibody. Detection was performed with NBT/BCIP (Roche) as substrates.

### Site-directed mutagenesis of PCX *N*-glycosylation residues

*N*-glycosylation sites were predicted via ExPASy tools [71]. Three putative *N*-glycosylation sites (Asn–Xaa–Ser/Thr motif), Asn^208^, Asn^326^, and Asn^442^, were predicted and mutated by the Quick Change II Site-Directed mutagenesis Kit (Stratagene, USA); Asn^208^, Asn^326^, and Asn^442^ were mutated into Asp (D) and Gln (Q). The primers used are listed in Additional file 9.

### Protein structure modeling

Modeling of the PCX structure was performed with the program SWISS-MODEL (http://swissmodel.expasy.org/) [72]. The template was selected based on sequence alignment. The *P. chrysosporium* cellobiohydrolase Cel7D (CBH58) sequence (UniProtKB code Q7LIJ0) showed 82% sequence identity with PCX. The protein-lactose complex structure of CBH58 was solved in 2005 (PDB code 1Z3V) [73] and was defined as the template. The model structure of PCX (Additional file 5) was presented with the molecular visualization system PyMOL.

## Abbreviations

4-MUC: 4-methyl umbelliferyl cellobiose; CBP: Consolidated bioprocess; CMC: Carboxylmethyl cellulose; dye-CMC: Dye-carboxylmethyl cellulose; pNPG: *p*-nitrophenyl-β-D-glucopyranoside; SDS–PAGE: Sodium dodecyl sulfate–polyacrylamide gel electrophoresis; SHF: Separate hydrolysis and fermentation; SSF: Simultaneous saccharification and fermentation; SSCF: Simultaneous saccharification and co-fermentation.

## Competing interests

The authors declare that they have no competing interests.

## Authors’ contributions

TYW, HLC, CJH designed, coordinated, conducted some of the experiments and/or analyses, and wrote the manuscript draft. HMS, WHL and MCS designed and supervised the research and revised the manuscript. HYC, HMK and YWC conducted the fungal culture, PCX cloning and mutagenesis. SKR and KYH conducted the yeast transformation and cellulase activity assay. PCH and ILW conducted the protein purification and related analyses. All the authors read and approved the final manuscript.

## Supplementary Material

Additional file 1 Cell density and doubling time of knockout strains with PCX expression in SC-URA medium. Click here for file

Additional file 2 **Time course of extracellular PCX activity in candidate strains.** The overnight cultures of these candidates were diluted into OD_600_ = 0.1 and grown in 50 ml SC-URA medium at 30°C for 24 hours. One milliliter of supernatant of each candidate was harvested every hour and total activity of extracellular PCX was then assayed using the 4-MUC method. 426GD(WT) is the plasmid without the PCX gene in the wild-type strain as a background control. Click here for file

Additional file 3 **Extracellular PCX activity in 50 ml supernatants of different hosts as measured by 4-MUC assay.** WT: BY4741; *∆*M: *mnn10* null mutant; *∆*V: *vps21* null mutant; *∆*P: *pmt5* null mutant; *alg1-1*: *ALG1* deficient mutant; CBHI: exocellulase of *Trichoderma ressei*. Click here for file

Additional file 4 **PNGaseF-treated extracellular PCX activity in 50 ml supernatants of different hosts as measured by 4-MUC assay.** WT: BY4741; *∆*M: *mnn10* null mutant; *∆*V: *vps21* null mutant; *∆*P: *pmt5* null mutant; CBHI: exocellulase of *Trichoderma ressei*. The 1 mg extracellular total proteins were treated with 500 units PNGaseF (P0704, NEB), 0.1% NP-40 in 20 μl reaction volume for 4 h in 37°C and then interacted with 4-MUC substrate for 24 h at 30°C. *P < 0.05; ** *P* < 0.01. Click here for file

Additional file 5 **Model structure of PCX.** (A) Ribbon view of PCX model structure, which is a beta-sandwich that consists of two large antiparallel beta-sheets and a catalytic core. Cellulose as substrate is represented as a yellow-colored ball-and-stick model. (B) Another view of the cellulose-binding tunnel. Three possible *N*-glycosylation sites are shown as and red-colored ball-and-stick models. Click here for file

Additional file 6 **Map of pRS426-GD plasmid with PCX.** (A) alpha signal peptide; (B) non-alpha signal peptide. Click here for file

Additional file 7 Extracellular PCX activity at different pHs and reaction temperatures as measured by 4-MUC assay. Click here for file

Additional file 8 **Relative copy number of expression plasmids and total 4-MUC activity.** The copy number of the *Ampicillin* gene from the pRS426GD plasmid was normalized with the *Adh1* gene in the BY4741 genome. The yeast cells were harvested and the genomic DNA was extracted by Fast ID Genomic DNA Extraction Kit (Genetic ID, Fairfield, IA) following the manufacturer’s instructions. The relative amount of each gene was normalized to that of the *Act1* gene (∆Ct) and quantified with the ∆∆Ct relative quantification method and the relative ratio was determined following the guidelines issued by Applied Biosystems. The amplification efficiency of each primer pair was tested by using 2-fold serial dilutions of the templates. The Q-PCR relative copy number ratio was determined by the ∆∆Ct value using the formula, the relative copy number ratio of (Ampicillin/ADH1) = 2^[−∆∆Ct]^, as suggested by Applied Biosystems, and the amplification efficiency of the target gene and the reference gene were approximately equal (*Adh1*: 95.4%, *Act1*: 87.7%, *Ampicillin*: 100%). Click here for file

Additional file 9 Primer sequences for site-directed mutagenesis. Click here for file
