# Supplementary material for: Systematic screening of glycosylation- and trafficking-associated gene knockouts in Saccharomyces cerevisiae identifies mutants with improved heterologous exocellulase activity and host secretion
Source: BMC Biotechnol. 2013 Sep 3;13:71. doi: 10.1186/1472-6750-13-71 (PMC3766678; doi:10.1186/1472-6750-13-71)
Supplement: Additional file 1 — Cell density and doubling time of knockout strains with PCX expression in SC-URA medium. [file 1472-6750-13-71-S1.pdf]

Additional file 1. Cell density and doubling time of knockout strains with PCX expression in SC-URA medium.

Strain	OD_{600} after 24 hours incubation	doubling time (minutes)
426GD(WT)	7.11±0.56	105
CBH1(WT)	6.14±0.47	111
PCX(WT)	5.04±2.06	191
PCX(ΔM)	4.13±0.92	166
PCX(ΔV)	6.39±0.34	113
PCX(ΔP)	7.28±0.60	101
PCX(ΔPΔV)	5.49±0.59	176
PCX(ΔMΔP)	4.58±1.62	139
PCX(ΔMΔV)	NA	-
