# Supplementary figures and images for: Systematic screening of glycosylation- and trafficking-associated gene knockouts in Saccharomyces cerevisiae identifies mutants with improved heterologous exocellulase activity and host secretion
Source: BMC Biotechnol. 2013 Sep 3;13:71. doi: 10.1186/1472-6750-13-71 (PMC3766678; doi:10.1186/1472-6750-13-71)

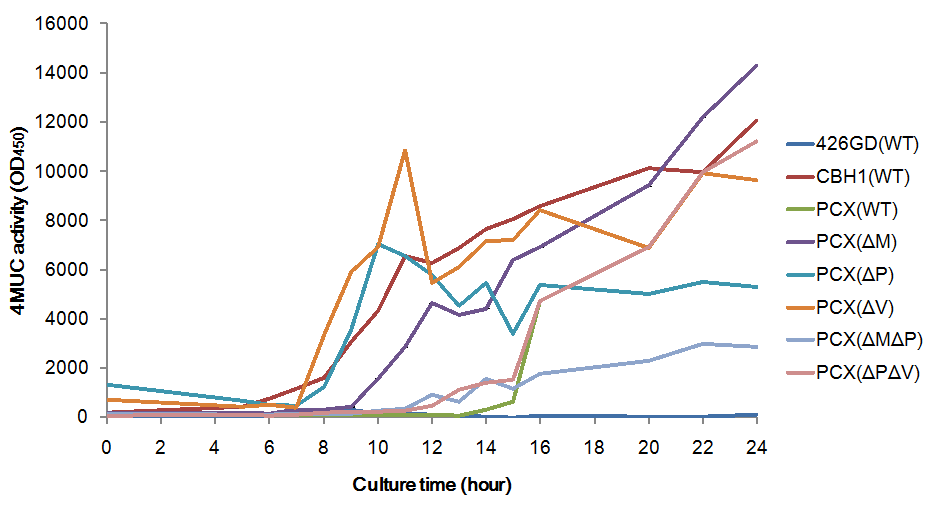

Supplement: Additional file 2 — Time course of extracellular PCX activity in candidate strains. The overnight cultures of these candidates were diluted into OD600 = 0.1 and grown in 50 ml SC-URA medium at 30°C for 24 hours. One milliliter of supernatant of each candidate was harvested every hour and total activity of extracellular PCX was then assayed using the 4-MUC method. 426GD(WT) is the plasmid without the PCX gene in the wild-type strain as a background control. [file 1472-6750-13-71-S2.tiff]

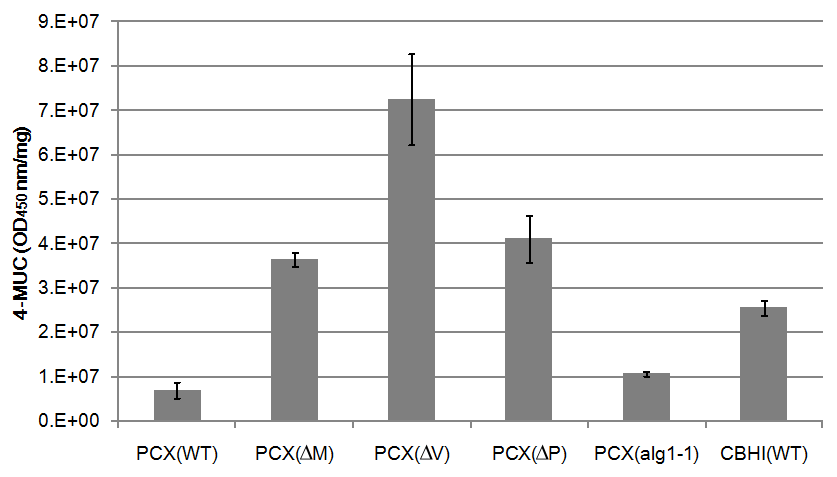

Supplement: Additional file 3 — Extracellular PCX activity in 50 ml supernatants of different hosts as measured by 4-MUC assay. WT: BY4741; ∆M: mnn10 null mutant; ∆V: vps21 null mutant; ∆P: pmt5 null mutant; alg1-1: ALG1 deficient mutant; CBHI: exocellulase of Trichoderma ressei. [file 1472-6750-13-71-S3.tiff]

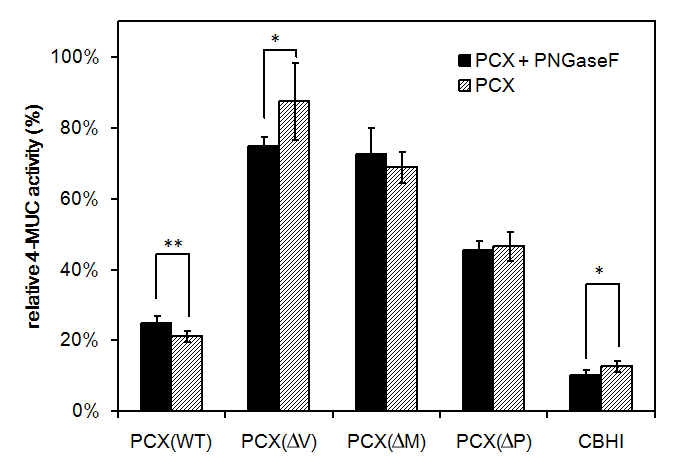

Supplement: Additional file 4 — PNGaseF-treated extracellular PCX activity in 50 ml supernatants of different hosts as measured by 4-MUC assay. WT: BY4741; ∆M: mnn10 null mutant; ∆V: vps21 null mutant; ∆P: pmt5 null mutant; CBHI: exocellulase of Trichoderma ressei. The 1 mg extracellular total proteins were treated with 500 units PNGaseF (P0704, NEB), 0.1% NP-40 in 20 μl reaction volume for 4 h in 37°C and then interacted with 4-MUC substrate for 24 h at 30°C. *P < 0.05; ** P < 0.01. [file 1472-6750-13-71-S4.tiff]

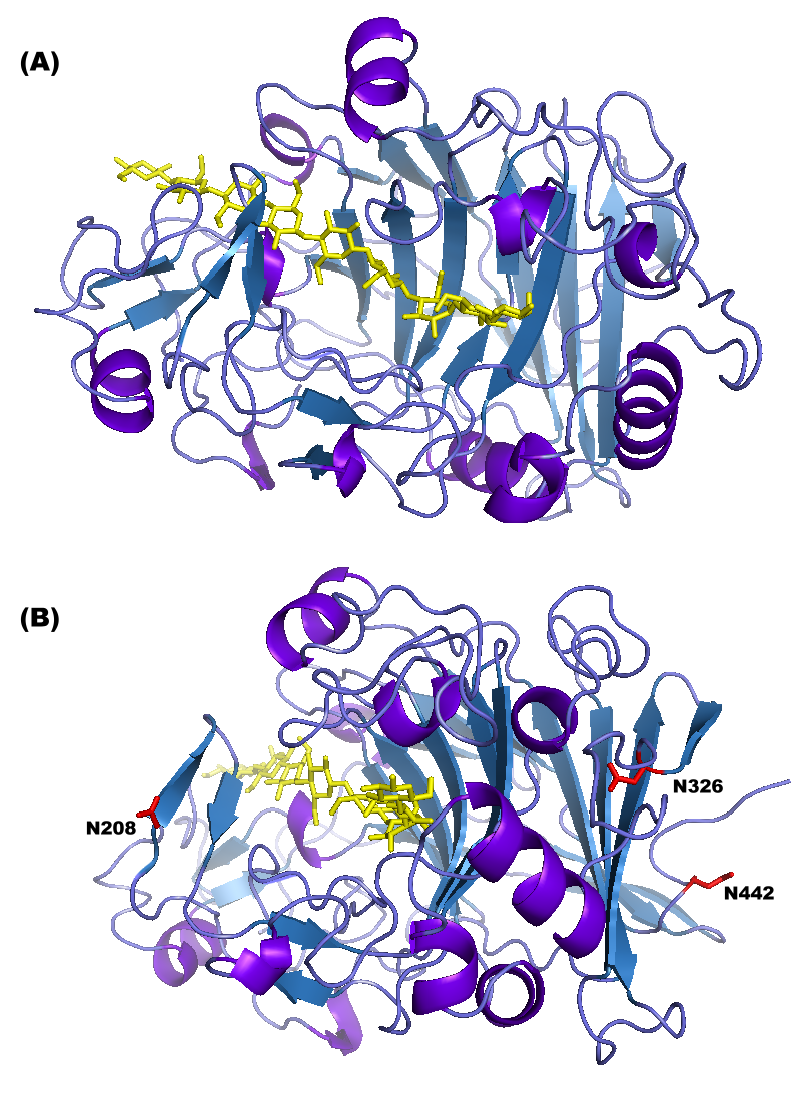

Supplement: Additional file 5 — Model structure of PCX. (A) Ribbon view of PCX model structure, which is a beta-sandwich that consists of two large antiparallel beta-sheets and a catalytic core. Cellulose as substrate is represented as a yellow-colored ball-and-stick model. (B) Another view of the cellulose-binding tunnel. Three possible N-glycosylation sites are shown as and red-colored ball-and-stick models. [file 1472-6750-13-71-S5.tiff]

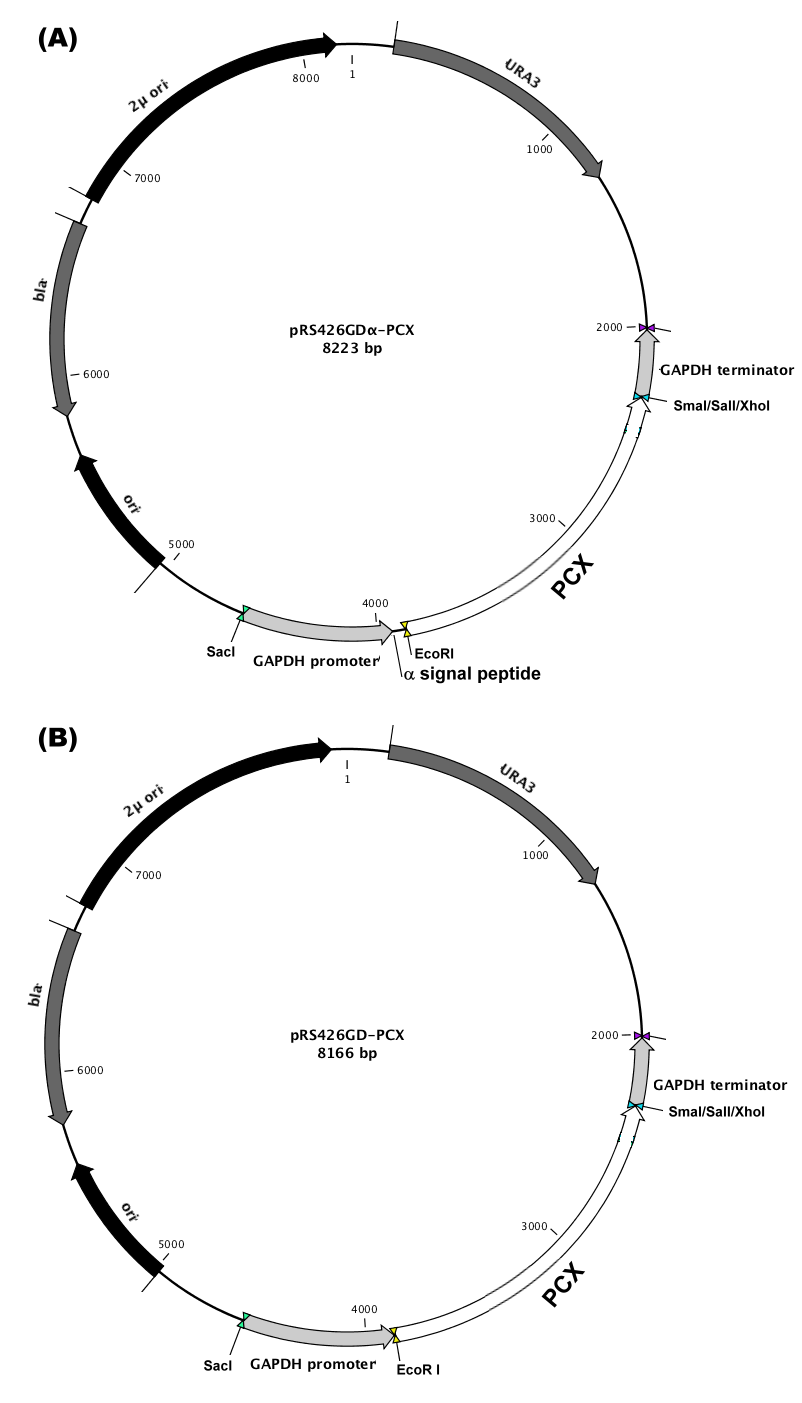

Supplement: Additional file 6 — Map of pRS426-GD plasmid with PCX. (A) alpha signal peptide; (B) non-alpha signal peptide. [file 1472-6750-13-71-S6.tiff]

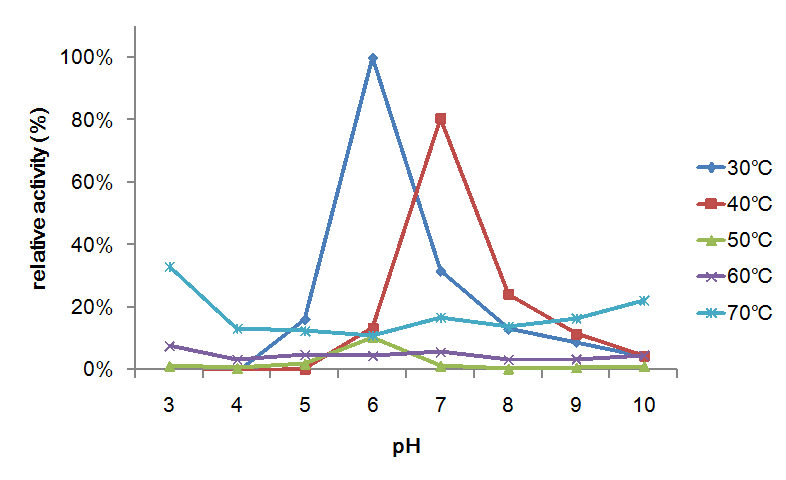

Supplement: Additional file 7 — Extracellular PCX activity at different pHs and reaction temperatures as measured by 4-MUC assay. [file 1472-6750-13-71-S7.tiff]

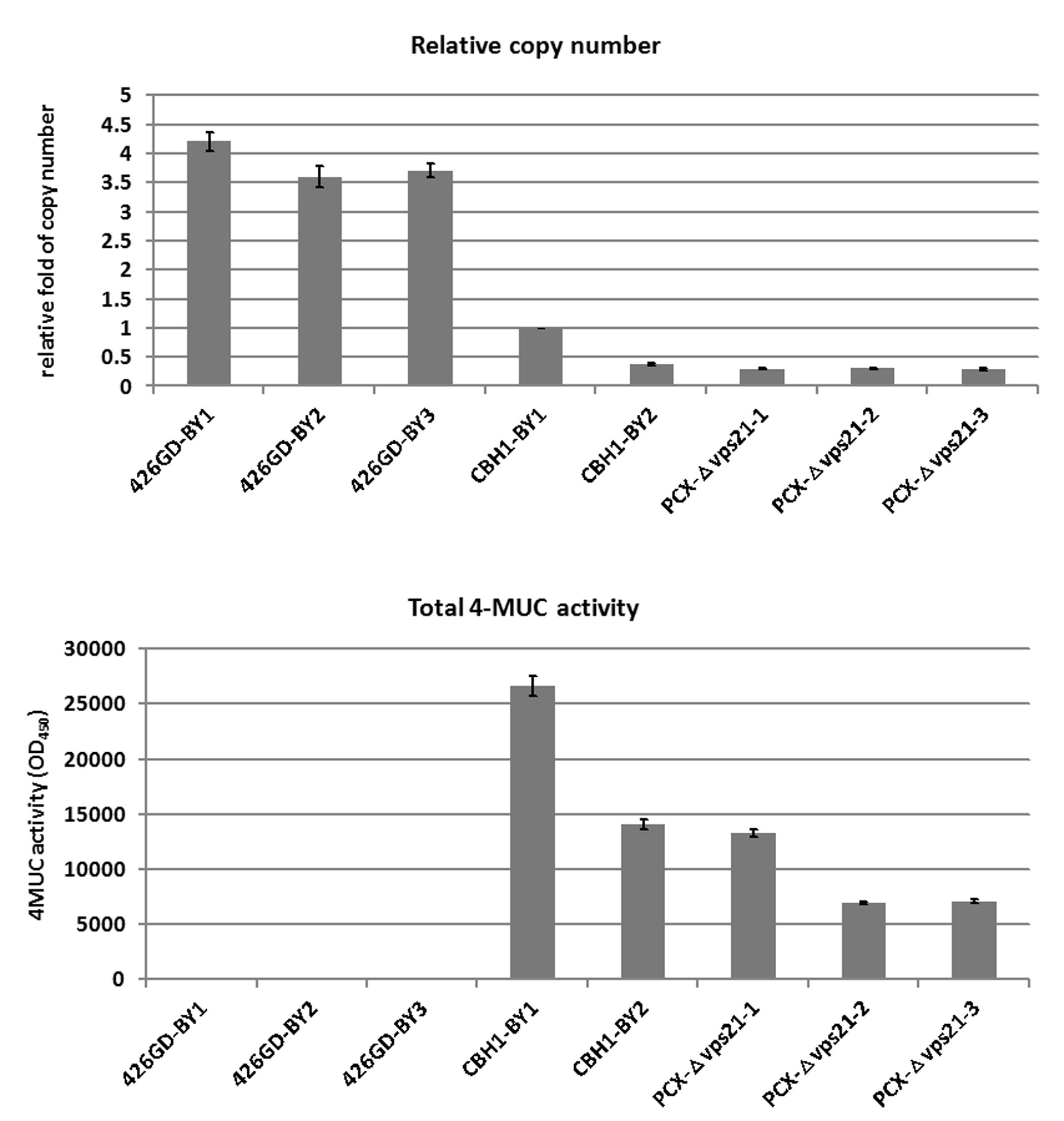

Supplement: Additional file 8 — Relative copy number of expression plasmids and total 4-MUC activity. The copy number of the Ampicillin gene from the pRS426GD plasmid was normalized with the Adh1 gene in the BY4741 genome. The yeast cells were harvested and the genomic DNA was extracted by Fast ID Genomic DNA Extraction Kit (Genetic ID, Fairfield, IA) following the manufacturer’s instructions. The relative amount of each gene was normalized to that of the Act1 gene (∆Ct) and quantified with the ∆∆Ct relative quantification method and the relative ratio was determined following the guidelines issued by Applied Biosystems. The amplification efficiency of each primer pair was tested by using 2-fold serial dilutions of the templates. The Q-PCR relative copy number ratio was determined by the ∆∆Ct value using the formula, the relative copy number ratio of (Ampicillin/ADH1) = 2[−∆∆Ct], as suggested by Applied Biosystems, and the amplification efficiency of the target gene and the reference gene were approximately equal (Adh1: 95.4%, Act1: 87.7%, Ampicillin: 100%). [file 1472-6750-13-71-S8.tiff]
