# Supplementary material for: Systematic screening of glycosylation- and trafficking-associated gene knockouts in Saccharomyces cerevisiae identifies mutants with improved heterologous exocellulase activity and host secretion
Source: BMC Biotechnol. 2013 Sep 3;13:71. doi: 10.1186/1472-6750-13-71 (PMC3766678; doi:10.1186/1472-6750-13-71)
Supplement: Additional file 9 — Primer sequences for site-directed mutagenesis. [file 1472-6750-13-71-S9.pdf]

Additional file 9. Primer sequences for site-directed mutagenesis.

substitution	name	Sequence
$Asn^{208} \rightarrow Gln$	N208Q-F	ACGTTGAGGGCTGGCAGGCCACCAGTGCCAACGCG
	N208Q-R	CGCGTTGGCACTGGTGGCCTGCCAGCCCTCAACGT
$Asn^{208} \rightarrow Asp$	N208D-F	ACGTTGAGGGCTGGGACGCCACCAGTGCCAACGCG
	N208D-R	CGCGTTGGCACTGGTGGCGTCCCAGCCCTCAACGT
$Asn^{326} \rightarrow Gln$	N326Q-F	CAGAATGGGAAGGTCATCCAGCAATCCTCCGTCAAGATCCCC
	N326Q-R	GGGGATCTTGACGGAGGATTGCTGGATGACCTTCCCATTCTG
$Asn^{326} \rightarrow Asp$	N326D-F	CAGAATGGGAAGGTCATCCAGGACTCCTCCGTCAAGATCCCC
	N326D-R	GGGGATCTTGACGGAGGAGTCCTGGATGACCTTCCCATTCTG
$Asn^{442} \rightarrow Gln$	N442Q-F	CATCAAGTTCGGCGACCTCCAGACCACGTACACCGGCACC
	N442Q-R	GGTGCCGGTGTACGTGGTCTGGAGGTCGCCGAACTTGATG
$Asn^{442} \rightarrow Asp$	N442D-F	CATCAAGTTCGGCGACCTCGACACCACGTACACCGGCACC
	N442D-R	GGTGCCGGTGTACGTGGTGTCGAGGTCGCCGAACTTGATG
